# Trend and equity of general practitioners’ allocation in China based on the data from 2012–2017

**DOI:** 10.1186/s12960-021-00561-8

**Published:** 2021-02-15

**Authors:** Qianqian Yu, Wenqiang Yin, Dongmei Huang, Kui Sun, Zhongming Chen, Hongwei Guo, Di Wu

**Affiliations:** 1 School of Management, Weifang Medical University, Baotong street No. 7166, Weifang, 261053 China; 2 Department of English, Weifang Medical University, Baotong street No. 7166, Weifang, 261053 China

**Keywords:** General practitioners, Resource allocation, Equity evaluation, Agglomeration analysis

## Abstract

**Background:**

General practitioners are the gatekeepers of the health of the residents. This study aims to evaluate the trend and equity of general practitioners**'** allocation from 2012 to 2017 in China and provide a reference for regional health planning and rational distribution of general practitioners.

**Methods:**

We extracted the data of general practitioners from 22 provinces, 5 autonomous regions, and 4 municipalities of mainland China. The population and geographical area were taken from the China Statistical Yearbook. The general practitioners**'** data were taken from the China Health Statistical Yearbook. Lorenz curve, Gini coefficient, and agglomeration degree were used to analyze the data.

**Results:**

The number of general practitioners was 252,717 in 2017, which equates to 1.82 per 10,000 residents, and accounts for 7.45% of the total number of practicing (assistant) doctors. From 2012 to 2017, the population-based Gini coefficient for general practitioners reduced from 0.31 to 0.24, while the geographical area-based Gini coefficient remained unchanged at 0.73. The agglomeration degree based on population increased from 0.72 to 0.73 in the western region including Tibet (0.403) and Shaanxi (0.513). Moreover, in the eastern region the agglomeration degree reduced from 1.477 to 1.329. In the middle region it rose from 0.646 to 0.802. The agglomeration degree based on the geographical area in the western region increased from 0.270 to 0.277 while the values in Tibet, Qinghai, Xinjiang were less than 0.1. In the eastern region, it reduced from 1.447 to 1.329. It increased from 1.149 to 1.423 in the middle region.

**Conclusions:**

The number of general practitioners has increased significantly in China. It has a fair allocation based on population. However, the equity based on geographical area is low and uneven in different regions with large regional differences. In the western region, there is an allocation shortage with respect to population and geographical area. Concerned departments should establish and improve the incentive and performance appraisal mechanisms of general practitioners. The Internet + should be used to empower their service capacity and efficiency. The educational input should be increased for the western region and government should encourage the eastern region to support the western region.

## Introduction

As per the World Health Organization, access to health is everyone's right, and everyone has the right to access essential medical and health services [1]. Based on the theory of general practice, general practitioners can treat 80 to 90% of the common diseases, frequently-occurring diseases, senile diseases, and chronic diseases in primary medical institutions. These general practitioners are the ones who provide continuous, individual, and comprehensive primary medical and health services and are known as the gatekeepers of residents' health [2]. Therefore, the equity of general practitioner resource allocation is of great significance to the residents' access to essential medical and health services. This equity consists of two aspects. First is the population allocation equity that is, the number of human resources for health per thousand people in different regions should be equal. The second is the geographical distribution equity that is the public equity of the spatial distance of human resources for health. The number of human resources for health in each square kilometer of different regions should be equal [3]. China has an aging population [4] and is experiencing an increase in chronic non-communicable diseases [5] and the total health expenditure [6], overcrowding in major hospitals, and a dearth of primary medical institutions [7].

General practitioners play a critical role in coping with the current challenges, promoting the implementation of the hierarchical medical system, and maintaining and promoting the health of the people [8–11]. However, in 2017, the total number of general practitioners in China was 252,717, accounting for 7.45% of the total number of practicing (assistant) physicians. The number of general practitioners per 10,000 residents was 1.82, which was below the target of 2–3; there also existed an unbalance of allocation. Therefore, the Chinese Government attached great importance to the development of general practitioners. We have issued some policy document measures related to the development, training, and incentive mechanisms of general practitioners to improve the quantity, quality, and work enthusiasm of general practitioners [12–15].

The research on the equity of general practitioners' allocation provides essential evidence for optimizing the allocation of resources. Investigators use different methods to evaluate and analyze the equity of general practitioners corresponding to different times. For instance, the researchers plotted Lorenz curves and calculated Gini, Atkinson, and Robin Hood indices and decile ratios to investigate the degree of inequality of general practitioners in Albania [16]. Geographic Information System network analysis was used to analyze the accessibility of general practitioners in Munich [17]. Robin Hood Index was used to analyze the equity of general practitioners' distribution in Australia [18, 19].

In China, the study on equity analysis of general practitioners' allocation includes two aspects. The first aspect is to analyze the equity of general practitioners' resource allocation in China in general. For instance, the Lorenz curve and Gini coefficient were used to analyze the allocation of general practitioners' resources in 2012 [20]. Moreover, the Theil index was used to analyze the equity of allocation of general practitioners in China from 2012 to 2016 [21]. Similarly, the Lorenz curve, Gini coefficient, and Theil index were used to evaluate the equity of general practitioners' resources in population, economy, and geographical distribution from 2012 to 2014 in China [22]. The second aspect is to analyze the equity of the allocation of general practitioners at the regional level in China. For example, the concentration index and Gini coefficient were used to analyze the equity of general practitioners' allocation in Guangxi from 2013 to 2016 [23]. Moreover, the Gini coefficient and Theil index were used to study the current situation and the equity trend of general practitioners' allocation in Shandong province from 2013 to 2016 [24]. Similarly, the maximum/minimum value (multiple), relative difference coefficient, Gini coefficient, and difference index were used to analyze the difference and fairness of general practitioner allocation in each district and county in Beijing [25].

As described above, most studies have used the Lorenz curve, Gini coefficient, and Theil index-based methods to analyze the equity of general practitioners' allocation in China as a whole or in a particular area of China. However, these methods have some limitations. Lorenz curve and Gini coefficient can reflect the overall degree of fairness but are unable to evaluate the unfairness of the regions [26]. The Thiel index can distinguish regional inequality, but it does not take into account the influence of geography [27]. The agglomeration degree analysis of health resources proposed in this study not only takes into account the equity of resource allocation of population distribution and geographical distribution but also analyzes the regional equity differences [28]. Besides, previous studies are usually limited to the analysis of the equity of general practitioners' resource allocation at the national level or in a particular region at a specific time and less focused on the comprehensive analysis of the equity of resource allocation at the national level and in specific regions. Nevertheless, it is necessary for the administrative department to timely adjust and optimize resource allocation. For this reason, our study aimed to analyze the current situation, trend, and equity of general practitioners' allocation from 2012 to 2017 in China. We used the Gini coefficient to discuss the equity of overall general practitioners' allocation according to the geographical area and population. Further, we used agglomeration analysis to evaluate the equity of different areas according to the geographical area and population. This study aimed to act as a guide for the Government to further optimize the allocation of the general practitioners’ resources in China.

## Materials and methods

### Data sources

This study used the data of general practitioners from 31 provinces, autonomous regions, and municipalities (except Taiwan, Hong Kong Special Administrative Region, and Macao Special Administrative Region) as research materials. We obtained the year-end population (population) and jurisdiction area (geography) of each region from the China Statistical Yearbook (2013–2018) [29–34]. General practitioners' data were obtained from the China Health Statistical Yearbook (2013–2018) [35–40]. The number of general practitioners in this study refers to the total number of practitioners (assistants) who either have registered as general practitioners or have obtained a general practitioner training certificate. We divided the Eastern region, central region, and the western region according to the China Health Statistics Yearbook 2018. The eastern region includes Beijing, Tianjin, Hebei, Liaoning, Shanghai, Jiangsu, Zhejiang, Fujian, Shandong, Guangdong, and Hainan (11 regions). The middle region includes Shanxi, Jilin, Heilongjiang, Anhui, Jiangxi, Henan, Hubei, and Hunan (8 regions). The western region includes Inner Mongolia, Chongqing, Guangxi, Sichuan, Guizhou, Yunnan, Tibet, Shaanxi, Gansu, Qinghai, Ningxia, and Xinjiang (12 regions).

### Data analysis

#### Lorenz curve and Gini coefficient

Lorenz curve is a graphical representation of income inequality or wealth inequality developed by American economist Max Lorenz in 1905. The more curved the Lorenz curve, the more unequal the income distribution.while the more flat the Lorenz curve, income distribution is more equal.Our study ranked 22 provinces, 5 autonomous regions, and 4 municipalities under their jurisdiction according to the number of general practitioners per capita. The Lorenz curve was then created according to the distribution of the service population by taking the cumulative percentage of general practitioners as the vertical coordinate and the cumulative percentage of the population as the horizontal coordinate. However, the 22 provinces, 5 autonomous regions, and 4 municipalities were ranked according to the number of general practitioners per square kilometer. The Lorenz curve distributed by the geographical area was created by taking the cumulative percentage of general practitioners as the vertical coordinate and the cumulative percentage of the population as the horizontal coordinate [26]. Calculated from the Lorenz curve, the Gini coefficient evaluates the equity of income distribution as defined by the American economist Albert Hirschman. Gini coefficient, whose value is between 0 and 1, is an important parameter that is used to comprehensively investigate the status of income distribution differences among residents on a global scale. The formula of the Gini coefficient is *G* = sigma (*X*_*i*_*Y*_*i*+ 1_– *X*_*i*+1_*Y*_*i*_), where *X*_*i*_ is the cumulative percentage of serving the population and geographical area of group *i* and *Y*_*i*_ is the cumulative percentage of GPS in group *i*. Gini coefficient of less than 0.2 means absolutely fair (best state). Gini coefficient of 0.2–0.3 means fair (good state), and the Gini coefficient of 0.3–0.4 means basic fair (normal state). Gini coefficient of 0.4–0.5 means unfair (alert state) and the Gini coefficient of more than 0.5 means very unfair (dangerous state). [41, 42]

### Agglomeration analysis

We used agglomeration analysis to measure the agglomeration degree of health resources in a particular region and the difference among different groups. The agglomeration analysis of general practitioners' resources was carried out in two dimensions, based on the geographical area and population. The formula of agglomeration degree based on geographical area is HRAD_*i*_ = (HR_*i*_/A_*i*_)/(HR_*n*_/A_*n*_). HR_*i*_ represents the number of general practitioners in the *i* region, and the HR_*n*_ represents the total number of general practitioners in China. A_*i*_ represents the land area in the *i* region, and A_*n*_ represents the land area in China. The formula of agglomeration degree based on population is HRAD_*i*_/PAD_*i*_ = (HR_*i*_/P_*i*_)/(HR_*n*_/P_*n*_). PAD_*i*_ represents the population agglomeration degree in the *i* region. HR_*i*_ and HR_*n*_ have the same meaning as above. P_*i*_ represents the number of population in the *i* region, and the P_*n*_ represents the total number of population in China [43].

Evaluation criteria: If the agglomeration degree based on geographical area is 1, the allocation of general practitioners is absolutely equitable in this region. If the agglomeration degree based on geographical area is close to 1, the equity of distribution in terms of the geographical area is better. Similarly, if the agglomeration degree based on population size is 1, the allocation of general practitioners is absolutely equitable in this region. If the agglomeration degree based on population size is close to 1, the equity of distribution in terms of population is better [43].

## Results

We analyzed the trend and equity of general practitioners' allocation in 2012–2017 in China from the national and area level, and the analyzed parameters included the Gini Coefficient, Lorenz curve and agglomeration degree.We also explored the proportion and registration rate of general practitioners.

- 1. The trend of General Practitioners in China from 2012 to 2017

*Status of general practitioners in 2017:* The number of general practitioners in China is 252,717, among which 139,473 were located in the east, 63,269 in the central region, and 49,975 in the west, accounting for 55.19%, 25.04%, and 19.78%, respectively. The average number of general practitioners per 10,000 population in China was 1.82. Moreover, the average number of general practitioners per 10,000 population was 2.42 in the eastern region, 1.46 in the middle region, and 1.33 in the western region. According to the analysis of every province, autonomous region, and municipality directly under the Central Government, the number of general practitioners per 10,000 population in Tibet and Shaanxi was less than 1, while that in Zhejiang, Shanghai, Beijing, and Jiangsu was more than 3. In Shandong, Henan, and other provinces, autonomous regions, and municipalities, the value was below the national average. Tables 1 and 2 show the detailed results.

**Table 1:** Number of general practitioners in 2012–2017 in China

Area	2012	2013	2014	2015	2016	2017	Growth rate%
Total	109,794	145,511	172,597	188,649	209,083	252,717	130.17
Eastern region	66,401	84,464	96,979	104,015	116,537	139,473	110.05
Middle region	22,192	29,674	39,020	45,344	49,944	63,269	185.10
West region	21,201	31,373	36,598	39,290	42,602	49,975	135.72

**Table 2:** Number of general practitioners per 10,000 in 2012–2017 in China (person)

Area	2012	2013	2014	2015	2016	2017	Average growth rate%
Total	0.81	1.07	1.27	1.37	1.51	1.82	17.58
*Eastern region*	1.19	1.50	1.71	1.83	2.03	2.42	15.25
Beijing	3.93	4.00	3.82	3.81	3.87	3.96	0.15
Tianjin	0.77	0.97	1.07	1.39	1.54	2.41	25.63
Hebei	0.48	0.92	1.17	1.25	1.25	1.33	22.61
Liaoning	0.75	0.80	0.86	0.83	0.96	1.44	13.94
Shanghai	2.24	2.47	2.85	3.04	3.29	3.51	9.40
Jiangsu	1.90	2.22	2.48	2.61	3.15	3.43	12.54
Zhejiang	2.24	3.10	3.57	3.90	4.04	5.39	19.20
Fujian	0.69	0.96	1.13	1.33	1.49	1.76	20.60
Shandong	0.70	0.79	0.92	1.01	1.14	1.36	14.21
Guangdong	0.75	1.11	1.34	1.38	1.67	2.03	22.04
Hainan	0.47	0.65	0.81	0.96	1.08	1.22	21.02
*Middle region*	0.52	0.70	0.91	1.05	1.16	1.46	22.93
Shanxi	0.71	0.81	0.99	1.10	1.13	1.72	19.36
Jilin	0.45	0.61	0.84	1.05	1.24	1.89	33.24
Heilongjiang	0.54	0.75	0.97	1.13	1.17	1.19	17.12
Anhui	0.53	0.72	1.12	1.20	1.39	1.67	25.80
Jiangxi	0.46	0.54	0.66	1.73	0.79	1.14	19.90
Henan	0.50	0.68	0.89	1.09	1.27	1.63	26.66
Hubei	0.65	0.87	1.05	1.19	1.19	1.52	18.52
Hunan	0.39	0.59	0.75	0.90	0.96	1.03	21.44
*Western region*	0.58	0.86	0.99	1.06	1.14	1.33	18.06
Inner Mongolia	0.67	0.95	1.17	1.23	1.26	1.58	18.72
Guangxi	0.66	0.86	0.95	0.97	1.05	1.28	14.17
Chongqing	0.55	0.74	0.84	0.95	1.03	1.26	18.03
Sichuan	0.58	1.11	1.21	1.27	1.25	1.37	18.76
Guizhou	0.30	0.43	0.69	0.89	1.04	1.40	36.08
Yunna	0.69	0.91	0.87	0.90	0.99	1.09	9.58
Tibet	0.11	0.21	0.34	0.50	0.61	0.73	46.01
Shaanxi	0.49	0.53	0.73	0.56	0.72	0.93	13.67
Gansu	0.54	0.82	1.05	1.27	1.45	1.46	22.01
Qinghai	0.81	1.31	1.51	1.63	1.67	2.06	20.52
Ningxia	0.40	0.60	0.71	1.85	0.97	1.36	27.73
Xinjiang	0.86	1.20	1.45	1.57	1.68	1.81	16.05

The total number of general practitioners in China has increased from 109,794 in 2012 to 252,717 in 2017. Moreover, the number of general practitioners has increased by 130.2% in the past five years. In 2012–2017, the average growth rate of general practitioners per 10,000 inhabitants in China was 17.58%, with the average growth rates in the eastern, central, and western regions were 15.25%, 22.93%, and 18.06%, respectively. From the analysis of every province, autonomous region, and municipalities under the Central Government, the average growth rates of Tibet, Guizhou, and Jilin ranked in the top three: 46.01%, 36.08%, and 33.24%, respectively. Tables 1 and 2 show the detailed results.

- 2. Gini Coefficient and Lorenz curve of Chinese general practitioners' allocation in 2012–2017

**Fig. 1:**
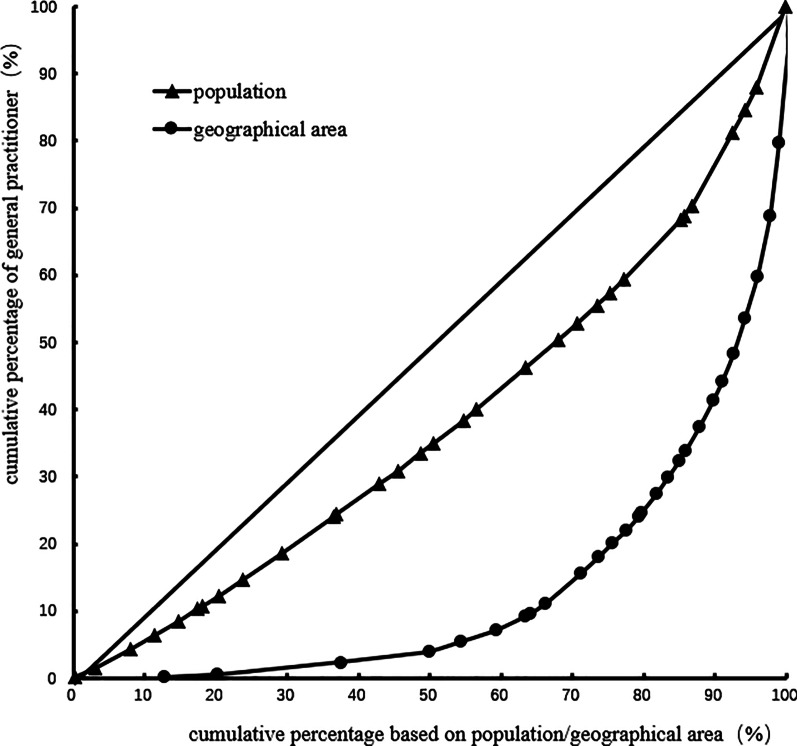
Lorenz curve for distribution of GPs in China in 2012

**Fig. 2:**
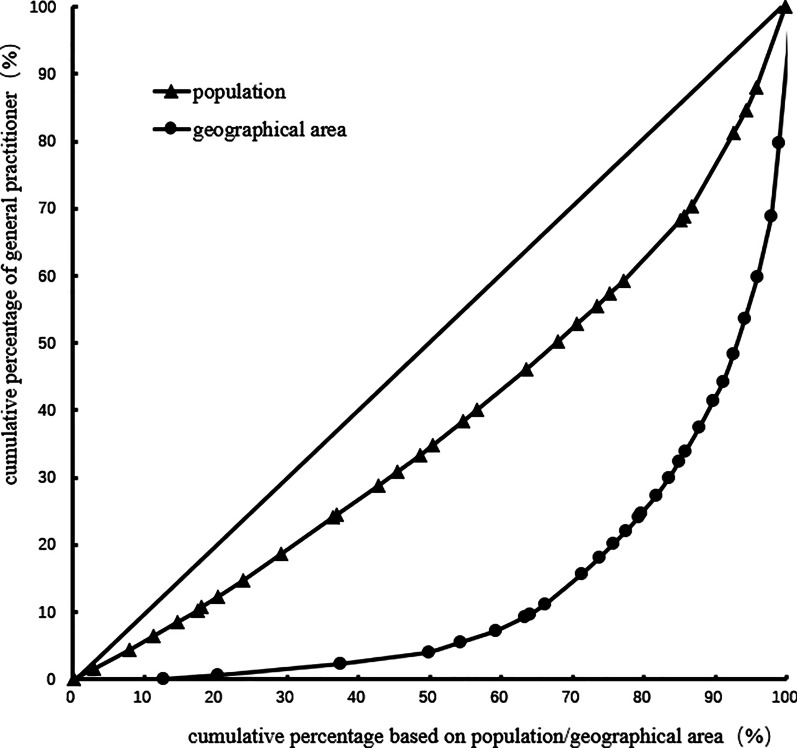
Lorenz curve for distribution of GPs in China in 2013

**Fig. 3:**
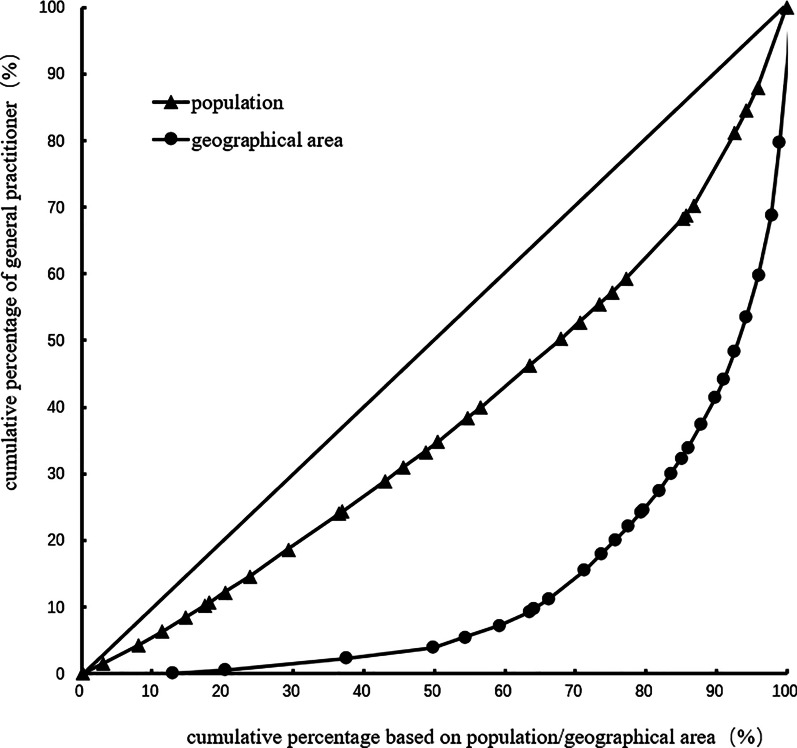
Lorenz curve for distribution of GPs in China in 2014

**Fig. 4:**
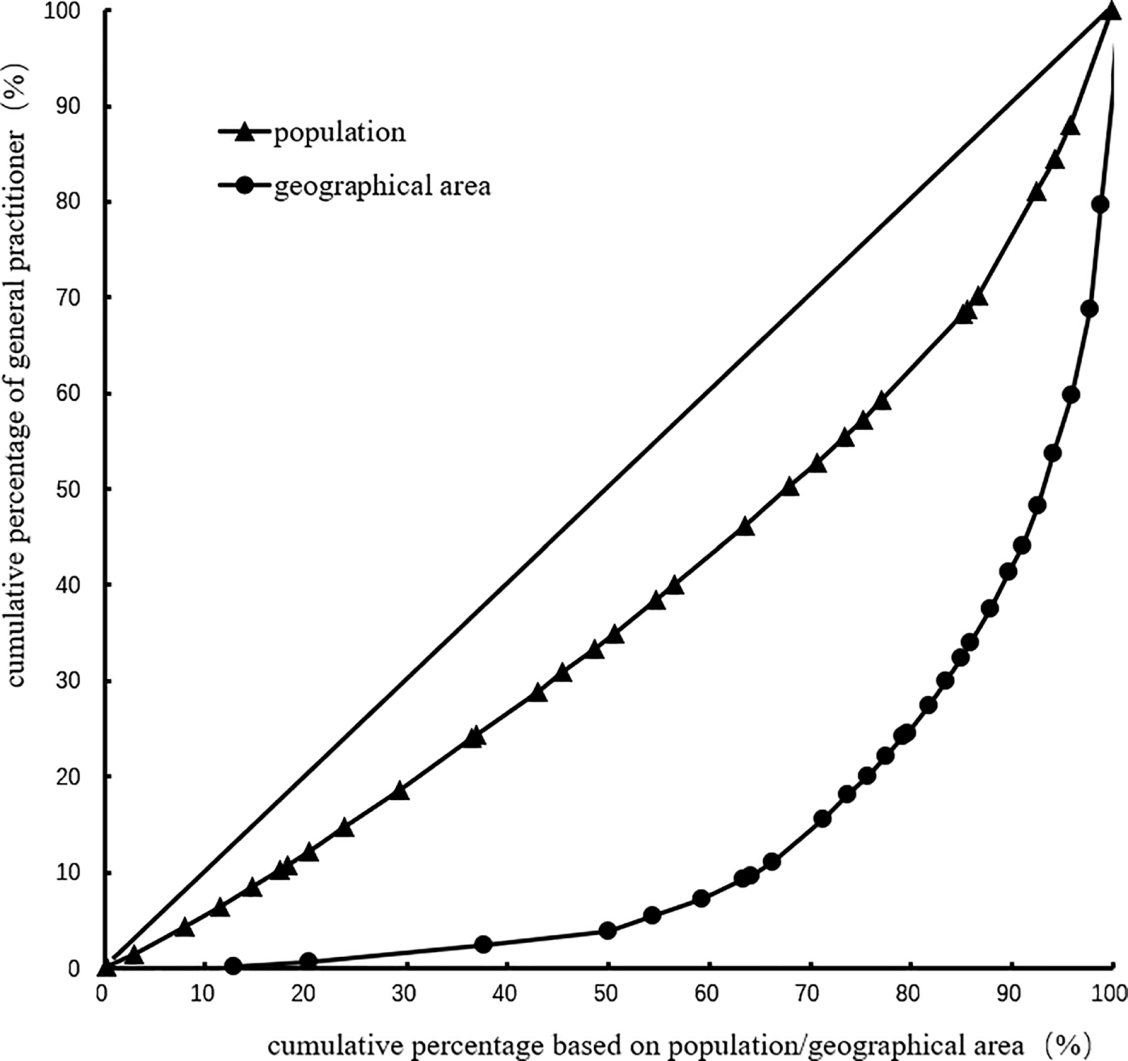
Lorenz curve for distribution of GPs in China in 2015

**Fig. 5:**
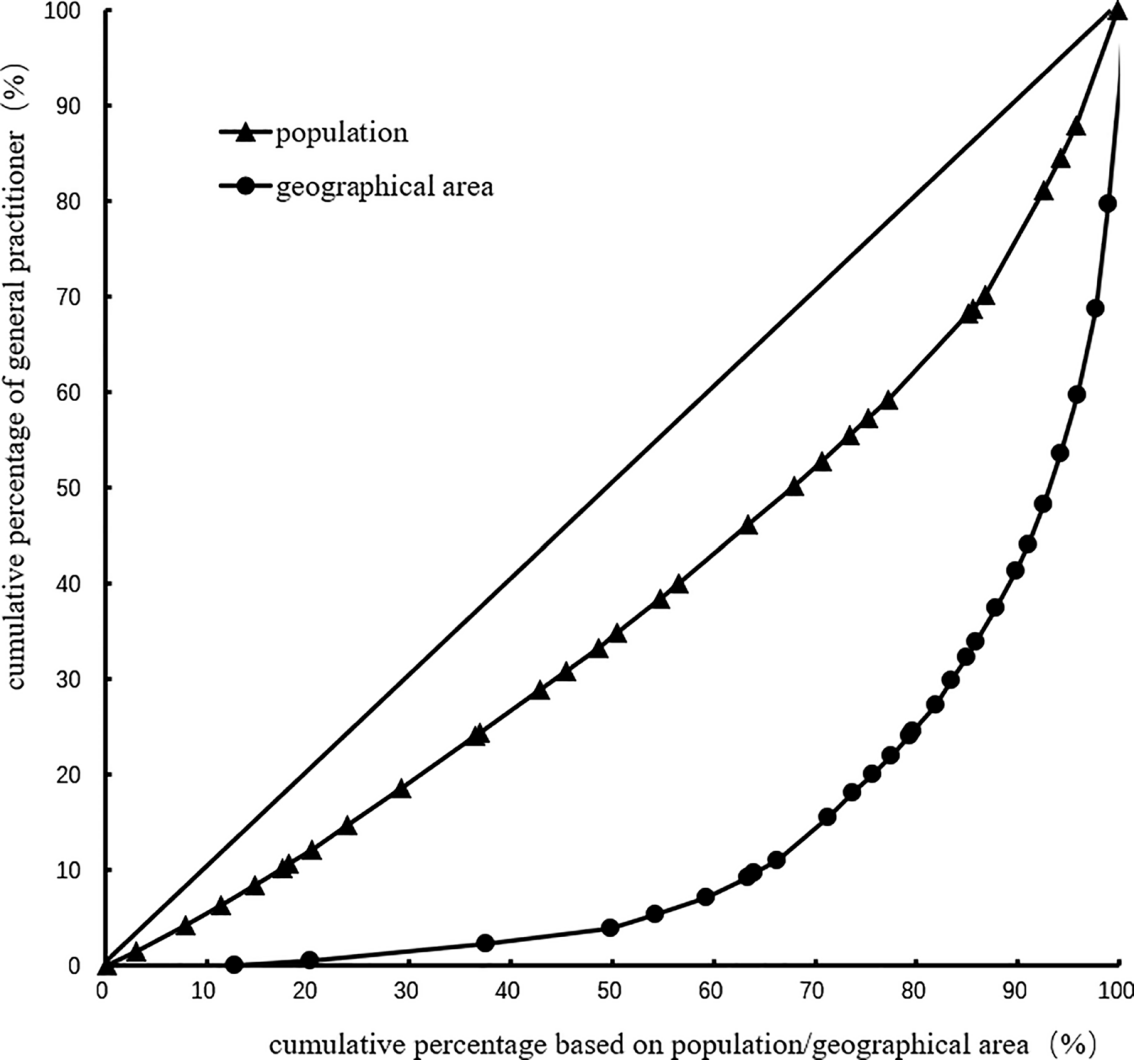
Lorenz curve for distribution of GPs in China in 2016

**Fig. 6:**
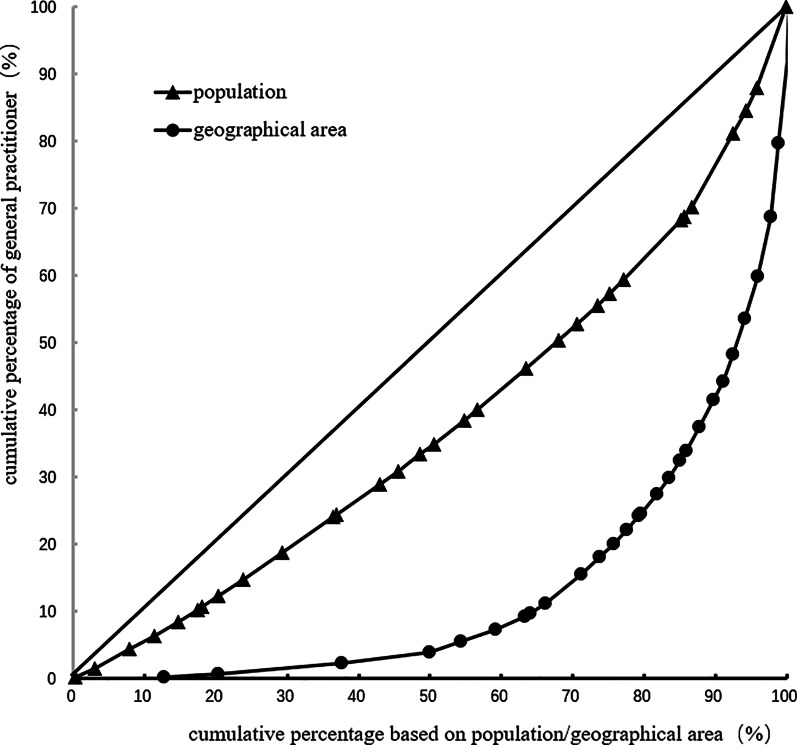
Lorenz curve for distribution of GPs in China in 2017

The Gini coefficient of general practitioners' allocation based on the population decreased from 0.31 to 0.24 in 2012–2017. Moreover, the Gini coefficient of general practitioners' allocation based on the geographical area was maintained at 0.72–0.73. These observations demonstrate that general practitioners' allocation in China based on the population has better equity, but that based on the geographical area has worse equity. Table 3 and Figs. 1, 2, 3, 4, 5, 6 show detailed results.

**Table 3:** Gini coefficient of Chinese general practitioners’ allocation in 2012–2017

Variable	2012	2013	2014	2015	2016	2017
Gini Coefficient (configured by population)	0.31	0.29	0.26	0.25	0.24	0.24
Gini Coefficient (configured by geographic area)	0.73	0.73	0.72	0.72	0.72	0.73

- 3. Agglomeration analysis of general practitioners' allocation in China in 2012–2017
- (1) The overall situation in 2017

*Agglomeration analysis based on geographic area allocation*

From the analysis of the regional classification, the agglomeration degree was found to be 0.277 in the western region, which is far below the value of 1. This value is inequitable to the general practitioners' allocation. In the middle region, the agglomeration degree was 1.423, which is slightly greater than 1, so that indicates that it was relatively equitable of general practitioners' allocation. In the eastern region, the agglomeration degree was 4.900, which is far greater than 1, indicating a far excessive concentration of general practitioners' allocation. From agglomeration degrees of different provinces, autonomous regions, and municipalities analyzed, the agglomeration degree of Guangxi was equal to 1, indicating that it was absolutely equitable. The agglomeration degree of certain areas was less than 1: Sichuan (0.89), Shanghai (0.66), and Ningxia Hui (0.53). This indicated the inequity of general practitioners' allocation based on the geographical area. Similarly, Tibet (0.01), Qinghai (0.06), Xinjiang (0.1), Inner Mongolia (0.13), Gansu (0.34), Heilongjiang (0.36), Yunnan (0.51), Ningxia (0.53), Shaanxi (0.66), and other areas have low agglomeration degrees, which indicated high inequity of general practitioners' allocation based on the geographical area [6]. The agglomeration degrees of general practitioners in other provinces and cities were greater than 1. Some of these values exceeded 10–51.198 (Shanghai), 19.899 (Beijing), and 11.968 (Tianjin), 10.970 (Zhejiang), indicating that the general practitioners' allocation was over-concentrated based on the geographical area. Table 4 shows the detailed data.

**Table 4:** Trend of agglomeration degree of general practitioners' allocation in 2012–2017 in China

Area	2012		2013		2014		2015		2016		2017		Growth rate%	
	HRADi	HRADi /PADi	HRADi	HRADi /PADi	HRADi	HRADi /PADi	HRADi	HRADi /PADi	HRADi	HRADi /PADi	HRADi	HRADi /PADi	HRADi	HRADi /PADi
*Eastern region*	5.370	1.477	5.154	1.405	4.989	1.359	4.896	1.332	4.949	1.344	4.900	1.329	− 8.75	− 10.02
Beijing	43.382	4.97	34.03	3.74	27.882	3.027	25.66	2.775	23.523	2.557	19.899	2.177	− 54.13	− 56.2
Tianjin	8.046	0.997	7.911	0.907	7.581	0.847	9.168	1.010	9.272	1.017	11.968	1.324	48.74	32.8
Hebei	1.618	0.595	2.352	0.858	2.544	0.927	2.503	0.911	2.275	0.828	2.015	0.733	24.54	23.19
Liaoning	1.952	0.93	1.566	0.748	1.419	0.682	1.246	0.603	1.301	0.634	1.61	0.79	− 17.52	− 15.05
Shanghai	73.877	2.797	62.38	2.307	61.139	2.262	59.39	2.218	58.06	2.177	51.198	1.932	− 30.7	− 30.93
Jiangsu	12.29	2.353	10.86	2.079	10.246	1.966	9.893	1.904	10.78	2.08	9.772	1.889	− 20.49	− 19.72
Zhejiang	10.153	2.766	10.66	2.898	10.354	2.826	10.43	2.845	9.823	2.67	10.97	2.962	8.05	7.09
Fujian	1.829	0.86	1.933	0.9	1.933	0.897	2.102	0.972	2.142	0.988	2.113	0.97	15.53	12.79
Shandong	3.749	0.867	3.219	0.741	3.157	0.726	3.195	0.734	3.305	0.756	3.261	0.746	− 13.02	− 13.96
Guangdong	3.863	0.932	4.319	1.034	4.458	1.064	4.235	1.004	4.686	1.103	4.801	1.119	24.28	20.06
Hainan	1.04	0.592	1.081	0.606	1.144	0.639	1.258	0.700	1.279	0.711	1.216	0.673	16.92	13.68
*Middle region*	1.149	0.646	1.159	0. 65	1.285	0.722	1.366	0.767	1.358	0.764	1.423	0.802	23.85	24.15
Shanxi	1.424	0.876	1.245	0.762	1.284	0.786	1.304	0.798	1.223	0.75	1.545	0.947	8.5	8.11
Jilin	0.574	0.552	0.591	0.571	0.682	0.662	0.785	0.765	0.829	0.819	1.04	1.039	81.18	88.22
Heilongjiang	0.385	0.669	0.403	0.704	0.439	0.771	0.465	0.826	0.432	0.775	0.361	0.652	− 6.23	− 2.54
Anhui	1.992	0.659	2.034	0.67	2.705	0.888	2.673	0.873	2.827	0.921	2.828	0.917	41.97	39.15
Jiangxi	1.09	0.572	0.96	0.502	1.006	0.527	1.012	0.53	1.002	0.524	1.199	0.627	10	9.62
Henan	2.472	0.62	2.539	0.638	2.796	0.705	3.154	0.795	3.335	0.841	3.541	0.896	43.24	44.52
Hubei	1.765	0.804	1.79	0.813	1.822	0.83	1.908	0.868	1.734	0.789	1.833	0.836	3.85	3.98
Hunan	1.066	0.483	1.224	0.549	1.327	0.595	1.471	0.658	1.413	0.632	1.263	0.564	18.48	16.77
*Western region*	0.270	0.722	0.302	0.801	0.297	0.787	0.291	0.771	0.285	0.753	0.277	0.729	2.59	0.97
Inner Mongolia	0.124	0.834	0.132	0.889	0.138	0.929	0.133	0.895	0.123	0.834	0.128	0.867	3.23	3.96
Guangxi	1.136	0.82	1.122	0.800	1.06	0.755	1.000	0.710	0.986	0.698	1.003	0.707	− 11.71	− 13.78
Chongqing	1.732	0.69	1.751	0.689	1.706	0.67	1.774	0.694	1.742	0.678	1.782	0.692	2.89	0.29
Sichuan	0.839	0.715	1.219	1.036	1.124	0.956	1.088	0.923	0.979	0.829	0.887	0.752	5.72	5.17
Guizhou	0.513	0.367	0.566	0.403	0.764	0.546	0.91	0.65	0.969	0.691	1.082	0.77	110.9	109.8
Yunna	0.713	0.855	0.713	0.85	0.58	0.69	0.554	0.659	0.552	0.657	0.506	0.602	− 29.03	− 29.59
Tibet	0.002	0.138	0.004	0.201	0.005	0.272	0.007	0.362	0.008	0.404	0.008	0.403	300	192
Shaanxi	0.776	0.601	0.635	0.491	0.749	0.582	0.526	0.408	0.611	0.475	0.661	0.513	− 14.82	− 14.64
Gansu	0.285	0.668	0.326	0.763	0.354	0.829	0.396	0.928	0.407	0.956	0.341	0.801	19.65	19.91
Qinghai	0.056	1.003	0.069	1.226	0.068	1.198	0.068	1.191	0.063	1.107	0.065	1.131	16.07	12.76
Ningxia	0.342	0.502	0.389	0.561	0.395	0.564	0.433	0.616	0.452	0.641	0.53	0.747	54.97	48.8
Xinjiang	0.101	1.075	0.108	1.122	0.111	1.147	0.114	1.145	0.111	1.109	0.101	0.997	0.000	− 7.26

*Agglomeration analysis by population allocation*

The agglomeration degrees in the eastern, middle, and western regions were 1.33, 0.80, and 0.73, respectively. Our data shows that the agglomeration degree based on the population allocation of the middle and western regions was less than 1, indicating that the general practitioners' resources allocation based on population was insufficient. The ratio of the eastern region was higher than 1, indicating that the general practitioners were too concentrated based on the population allocation. From the perspective of different provinces, autonomous regions, and municipalities, there was a difference in the fairness of population allocation. The resource allocation of general practitioners in Xinjiang (1) was absolutely fair. The agglomeration degree in Fujian (0.97), Jilin (1.04), and Shanxi (0.95) approached 1, indicating that their general practitioners' resources were fair according to the population allocation. However, the agglomeration degree in Zhejiang (2.96), Beijing (2.18), and Shanghai (1.93) were in the top three and far greater than 1, indicating that the general practitioners' resources in these areas were too concentrated according to the population allocation. The agglomeration degree of 23 provinces and municipalities such as Tibet (0.4), Shaanxi (0.51), and Hunan (0.56) were less than 1, indicating that the resources of general practitioners were relatively scarce, and the population allocation was insufficient. Table 4 shows the detailed data.

- (2) The trend of agglomeration degree of general practitioners' allocation in 2012–2017

*The trend of agglomeration degree by geographical area allocation*

From 2012 to 2017, the agglomeration degree decreased from 5.370 to 4.900 in the eastern region. Moreover, it increased from 1.149 to 1.423 in the middle region and decreased from 0.270 to 0.227 in the western region. From the analysis of agglomeration degrees in different provinces, autonomous regions, and municipalities, the agglomeration degree is relatively high in Shanghai, Jiangsu, Beijing, and Guangdong in 2012, but it has declined in recent years. For example, in Shanghai, it decreased from 73.877 in 2012 to 51.198 in 2017 (30.70% decrease). Similarly, in Beijing, it decreased from 43.382 in 2012 to 19.899 in 2017 (54.13% decrease). In Jiangsu, it decreased from 12.290 in 2012 to 9.772 in 2017 (20.49% decrease), and the resource allocation gradually became fair. The agglomeration degree in Tibet, Qinghai, Xinjiang, and Inner Mongolia was relatively low in 2012, and the fluctuations were not apparent in recent years. For example, in Tibet, the agglomeration degree increased from 0.002 in 2012 to 0.008 in 2017 (300% increase). In Qinghai, it increased from 0.056 in 2012 to 0.065 in 2017 (16.07% increase). From 2012 to 2017, the agglomeration degree of Xinjiang remained unchanged at 0.101. Further, in Inner Mongolia, it increased from 0.124 to 0.128 (3.23% increase). Table 4 shows the detailed data.

*The trend of agglomeration degree by population allocation*

From 2012 to 2017, the agglomeration degree in the eastern region was higher, which was from 1.477 and 1.329, and the general practitioners' allocation tended to be fair. The middle region allocation tended to be fair, which is between 0.646 and 0.802; however, in the western region, it was slightly lower (0.722–0.729). In the meantime, agglomeration degree also tended to be fair, and the general practitioners had better fairness according to the population allocation. From the analysis of different provinces, municipalities, and autonomous regions, the agglomeration degrees of Shanghai, Jiangsu, and Beijing in 2012 were relatively high, but these values declined over the years. For example, in Beijing, the agglomeration degree reduced from 4.97 in 2012 to 2.177 in 2017 (56.20% decrease). In Shanghai, it reduced from 2.797 in 2012 to 1.932 in 2017 (30.93% decrease). In Jiangsu, it reduced from 2.353 in 2012 to 1.889 in 2017 (19.72% decrease). The resource allocation gradually became fair. In 2012, the agglomeration degrees of Tibet, Guizhou, Hunan, Ningxia, and Jilin provinces were relatively low, but during the later years, they became more equitable. For example, in Tibet, the agglomeration degree increased from 0.138 in 2012 to 0.403 in 2017 (192.03% increase). In Guizhou, it increased from 0.367 in 2012 to 0.77 in 2017 (109.81% increase). In Hunan, it increased from 0.483 in 2012 to 0.564 in 2017 (16.77% increase). In Ningxia, it increased from 0.502 in 2012 to 0.747 in 2017 (48.80% increase). In Jilin, it increased from 0.552 in 2012 to 1.039 in 2017 (88.22% increase). We found that the general practitioners in China tended to be equitable based on population allocation. Table 4 shows the detailed data.

- 4. The proportion and registration rate of general practitioners in China in 2012–2017

According to the analysis of general practitioners in China, the proportion of general practitioners in practice (assistant) physicians increased from 4.20% in 2012 to 7.45% in 2017. The proportion of general practitioners in the western region was the lowest, which was only 6.34% in 2017. But, it was higher in the eastern region (9.09%). From the perspective of provinces, autonomous regions, and municipalities, Zhejiang (17.05%), Jiangsu (12.70%), Shanghai (12.50%), Tianjin (9.12%), and Beijing (9.10%) ranked in the top five in China (Table 5). However, Tibet (3.25%), Shaanxi (3.84%), Hunan (4.07%), Heilongjiang (5.08%), and Ningxia (5.09%) were the last five in China. Table 5 shows the detailed data

The registration rate of general practitioners was only 38.08% at the end of 2017; and 96,000 general practitioners were enrolled in general medicine major among 253,000 qualified general practitioners. The registration rate increased from 33.86% in 2012 to 38.08% in 2017. The registration rate had a fast growth in the eastern region (from 36.19% in 2012 to 41.75% in 2017). In the western region, the registration rate increased from 26.39% in 2012 to 30.82% in 2017. Moreover, the registration rate increased from 33.99% in 2012 to 35.73% in 2017 in the central region. From the perspective of provinces, autonomous regions, and municipalities, the top five in terms of registration rates were Shanghai (67.82%), Tibet (62.75%), Guangdong (54.32%), Beijing (54.30%), and Jiangsu (46.83%) in 2017. Between 2012 and 2017, the top five in terms of the growth in registration rates were Tianjin (192.07%), Shandong (100.71%), Qinghai (68.85%), Ningxia (64.66%), and Guangxi (56.44%). Table 6 shows the detailed data

**Table 5:** The proportion of general practitioners in practice (assistant) physicians in China in 2012–2017 (%)

Area	2012	2013	2014	2015	2016	2017
Total	4.20	5.21	5.21	6.21	6.55	7.45
*Eastern region*	5.65	6.70	6.70	7.62	8.09	9.09
Beijing	10.94	10.97	10.97	9.70	9.40	9.10
Tianjin	3.57	4.45	4.45	5.98	6.36	9.12
Hebei	2.44	4.48	4.48	5.56	5.28	5.22
Liaoning	3.27	3.40	3.40	3.47	3.82	5.42
Shanghai	9.54	10.28	10.28	11.67	12.18	12.50
Jiangsu	9.54	10.40	10.40	11.01	12.30	12.70
Zhejiang	9.43	12.32	12.32	13.68	13.42	17.05
Fujian	3.89	5.00	5.00	6.57	7.26	8.21
Shandong	3.38	3.33	3.33	4.18	4.64	5.13
Guangdong	3.99	5.59	5.59	6.54	7.54	8.80
Hainan	2.71	3.47	3.47	4.60	4.96	5.46
*Middle region*	2.85	3.60	3.60	4.98	5.27	6.34
Shanxi	2.92	3.35	3.35	4.45	4.55	6.76
Jilin	2.00	2.71	2.71	4.30	4.86	7.27
Heilongjiang	2.65	3.59	3.59	5.22	5.28	5.08
Anhui	3.47	4.38	4.38	6.83	7.65	8.63
Jiangxi	3.10	3.46	3.46	4.32	4.60	6.30
Henan	2.82	3.56	3.56	5.21	5.87	7.07
Hubei	3.44	4.30	4.30	5.13	4.95	6.09
Hunan	2.22	3.09	3.09	4.06	4.06	4.07
*Western region*	3.20	4.42	4.42	5.15	5.30	5.83
Inner Mongolia	2.82	3.83	3.83	4.80	4.79	5.67
Guangxi	3.96	4.85	4.85	5.10	5.28	6.20
Chongqing	3.14	3.97	3.97	4.71	4.83	5.64
Sichuan	2.86	5.17	5.17	5.73	5.59	5.82
Guizhou	2.10	2.70	2.70	4.96	5.38	6.64
Yunna	4.69	5.69	5.69	5.39	5.52	5.59
Tibet	0.84	1.29	1.29	2.59	3.09	3.25
Shaanxi	2.63	2.66	2.66	2.67	3.20	3.84
Gansu	3.23	4.69	4.69	6.68	7.15	6.81
Qinghai	3.88	5.73	5.73	6.97	7.26	7.93
Ningxia	2.00	2.74	2.74	3.56	3.83	5.09
Xinjiang	3.81	5.12	5.12	6.47	6.67	7.12

**Table 6:** General practitioners registration rate in 2012–2017 in China (%)

Area	2012	2013	2014	2015	2016	2017
Total	33.86	32.58	37.17	36.24	37.13	38.08
*Eastern region*	36.19	36.13	40.45	39.94	40.79	41.75
Beijing	50.46	50.33	51.34	52.53	52.32	54.3
Tianjin	15.89	19.41	28.24	30.83	44.61	46.41
Hebei	23.33	22.96	24.37	23.24	24.46	26.47
Liaoning	40.01	28.78	32.14	28.67	37.71	46.2
Shanghai	62.45	67.28	72.78	68.97	72.31	67.82
Jiangsu	36.63	34.83	38.12	38.32	35.03	46.83
Zhejiang	31.25	33.21	39.81	37.61	35.94	23.99
Fujian	28.72	25.76	28.52	28.7	30.35	35.42
Shandong	18.33	24.8	28.05	28.59	31.39	36.79
Guangdong	34.96	38.39	46.99	49.74	53.01	54.32
Hainan	42.99	42.59	45.74	46.06	47.26	45.37
*Middle region*	33.99	31.11	34.96	34.8	35.57	35.73
Shanxi	29.58	28.23	31.32	36.37	39.02	27.24
Jilin	33.23	34.58	36.67	32.27	37.2	31.5
Heilongjiang	31.76	24.78	28.28	29.31	30.13	31.49
Anhui	40.68	39.96	41.74	40.15	40.65	44.46
Jiangxi	33.49	33.35	35.89	34.56	33.48	23.97
Henan	33.12	26.42	29.75	31.38	32.01	39.41
Hubei	31.72	27.4	33.89	32.34	31.57	32.62
Hunan	37.57	37.81	41.98	41.12	41.65	40.91
*Western region*	26.39	24.39	30.84	28.11	28.93	30.82
Inner Mongolia	32.1	32.31	38.51	35.14	35.43	34.07
Guangxi	19.79	23.72	32.58	28.67	24.84	30.96
Chongqing	25.31	22.04	28.89	26.92	27.76	33.99
Sichuan	31.13	21.96	23.2	20.87	22.16	22.96
Guizhou	40.7	36.86	46.56	44.58	43.35	41.96
Yunna	19.99	18.24	22.19	18.07	20.69	27.26
Tibet	52.94	53.73	77.06	70.81	64.36	62.75
Shaanxi	18.2	19.46	36.71	28.17	25.64	23.56
Gansu	26.93	27.68	32.1	32.13	33.85	29.81
Qinghai	27.06	29.68	33.83	27.37	38.47	45.69
Ningxia	25.38	30.36	39.49	34.69	43.73	41.79
Xinjiang	31.38	29.08	35.49	34.12	34.83	35.21

## Discussion

Our study found the total allocation of general practitioners in China is insufficient and varies significantly among different regions. In western China, general practitioners are not allocated according to the population or geographical area, while in eastern China, there is an over-concentration of general practitioners. The discussion is divided into the following sections:

1. The number of general practitioners in China has been increasing significantly, but the total allocation is still insufficient, and the work of general practitioners is generally stressful

The Chinese Government has recently paid great attention to the development of general practice and the talent training of general practitioners. According to *Guidance of the State Council on the Establishment of a General Practitioner System* issued in 2011, there should be 2 ~ 3 qualified general practitioners for every 10,000 urban and rural residents by 2020. The China Health and Family Planning Yearbook (2013–2018) showed that the number of general practitioners increased from 109,794 in 2012 to 252,717 to 2017 in China. This increase in number is a good development. However, 7.45% of practicing (assistant) doctors were general practitioners by the end of 2017, which corresponded to 1.82 general practitioners per 10,000 population. In the western region, there were 1.33 general practitioners per 10,000 population [40]. The shortage of general practitioners leads to high work pressure, low job satisfaction, and easy job burnout [44–46].

Since the creation of general practitioners' education and training systems in China started late, the construction of the general practitioner system and theoretical research still has to be improved urgently, which is a challenging goal to achieve. Besides, general practitioners account for 30 ~ 60% of the total number of doctors in developed countries; however, in China, there is still a big gap [47].

Therefore, at first, the Government should continue to strengthen the training of general practitioners and build more reasonable and standardized general practitioners training bases to strengthen the development of general practitioners' teachers. Second, we should establish the general practitioners' job support system to adjust the working strength of general practitioners. We are using "Internet + " to assign general practitioners and provide services such as online signup, online consulting, and disease diagnosis. These strategies will reduce their work pressure and improve service efficiency [48–50].

2. The fairness of the number of general practitioners allocated according to population is fair, but that allocated according to the geographical area is low. There are regional differences between the eastern and the western regions

The Gini Coefficient of the number of general practitioners allocated according to the population in China decreased from 0.31 in 2012 to 0.24 in 2017, which is fair, but the Gini Coefficient allocated according to the geographical area was 0.72–0.73, which was very unfair. It shows that the fairness of general practitioners in population allocation is higher than that in geographical area allocation in China [20–22]. Chinese regional planning of health resources is mainly based on the allocation of health resources per 10,000 population, which is fair in population allocation and has shown a trend towards more equitable development in recent years. The analysis of the agglomeration degree reflects the differences between different regions. The agglomeration degree based on population was 1.33, 0.80, and 0.73 in the eastern, middle, and western regions, respectively. The resources of general practitioners in the middle region were insufficient based on the population allocation, and while those in the eastern region were too concentrated. The agglomeration degree based on geography was 0.27 in the western region that is relatively low in equity, 1.3 in the middle region that is fair, and 5.37 in the eastern region that is the excess of general practitioners' resources. They are very inequitable in the western region, based on the geographical area and population to allocate the general practitioners. There may be several reasons for these observations. For instance, these areas are mostly low-lying areas including plateaus and deserts. Further, the economic situation is poor, which reduces the attractiveness of the region. The per capita disposable income was 20,130.3 yuan ($3080.9) in the western region and 33,414.0 yuan ($5113.9) in the eastern region in 2017 [34]. Third, the western region is unfit for people to live because of the thin air, low pressure, and low oxygen content. Many projects of equalization of essential public health services involve door-to-door visits, and the size of the service area is also an essential factor for the smooth completion of the work, which needs to attract the attention of the Government and relevant departments [51, 52].

The general practitioners should play the role of health and expenditure gatekeepers in the western region. The Government should strengthen macro-control and increase the intensity of financial input to the western region. Firstly, the Government should encourage the hospitals in large and medium cities to help the grassroots hospitals to guarantee the development and echelon reserve of the general practitioners in the western region [53]. Secondly, the Government should formulate reasonable resource allocation standards and development plans based on the actual conditions in the western region and take measures to meet their needs. Besides, Chinese medical resources should be diverted to the western region to avoid widening inequality.

- 3. The equity of general practitioners' allocation has a big gap in different provinces and municipalities in China

Among 31 provinces, autonomous regions, and municipalities, agglomeration degree based on the population allocation is less than 0.2 in Tibet, Qinghai, Xinjiang, and Inner Mongolia. The value is less than 0.3 in Gansu and Heilongjiang and less than 0.7 in Yunnan, Ningxia, and Shaanxi. The number of general practitioners in the above areas is insufficient based on the population allocation. The agglomeration degree is less than 0.6 in Tibet, Shaanxi, Hunan, and other areas. The equity of the areas mentioned above is insufficient based on the geographical area allocation. However, the agglomeration degree based on the population or geographical area allocation is far greater than 1 in Shanghai, Beijing, Zhejiang, and other areas, which means that the allocation of general practitioners is excessively concentrated. Graduate medical students tend to work in the eastern region that has abundant resources and a developed economy. First, the Government should encourage graduate medical students to work in the area, which has an underdeveloped economy, sparse population, and a large geographical area. Second, the Government should comprehensively improve their remuneration, optimize promotion policies and give their children good eduation, which would attract more people to work in the western regions. Furthermore, the income of general practitioners should be improved [20]. Thirdly, the Government should vigorously develop medical education in areas with a shortage of talents to cultivate general medical talents, and actively carry out standardized training of resident physicians in the "5 + 3" mode and assistant general practitioners in the "3 + 2" mode. In the meantime, the Government should ensure they are well reimbursed for their work during the training and gradually increase the subsidy standard [54].

## Conclusion

In conclusion, it is crucial to analyze the equity of general practitioners' allocation to ensure equitable access to health resources for all. Our study indicated that the number of general practitioners in China had increased significantly, but the total allocation is still insufficient. China has a relatively fair allocation of general practitioners' resources based on population size, which is continuously improving. However, the equity distribution based on geographical area is low and has not changed much in recent years. The distribution of general practitioners in different regions is uneven with large regional differences. In the western region, there is a shortage of general practitioners in terms of population size and geographical area, while in the eastern region, there is an excessive concentration of resources. It is necessary to establish and improve the incentive mechanism and performance appraisal mechanism of general practitioners to improve the occupational attractiveness. Furthermore, we can introduce the Internet + to reduce the pressure and improve the efficiency of general practitioners. The Government should pay more attention to the western region for supporting the general practitioners' allocation, such as increasing financial support, developing medical education to achieve balanced development in different regions.

### Research limitations

Firstly, this study evaluated the equity of the general practitioners' allocation based on the hypothesis of resource homogeneity. The study does not distinguish the differences in service quality and serviceability between different general practitioners. Secondly, this study evaluated the equity of the general practitioners' allocation based on the population and geographical area. It does not analyze the economic conditions. However, the higher the level of economic development in a region, the higher the level of education and the living standards of the residents in that region. Therefore, the demand for human resources for health will increase accordingly.

## Data Availability

The data used for this manuscript from China Statistical yearbook and China health and family planning yearbook.

## References

[1] Organization WH. Research for Universal Health Coverage. The World Health Report 2013. Research for universal health coverage, 2013.

[2] Qin JM, Zhang LF, Lin CM, Zhang X, Zhang YC (2016). Scale and allocation of human resources in primary health care system in China after new medical reform. Chin Gen Pract.

[3] Doull L, Campbell F (2008). Human resources for health in fragile states. Lancet.

[4] Cheng ZQ, Ma JQ (2018). Evolution and measures of china's population ageing. Acad Exchange.

[5] Tang SL, John E, Long Q (2013). China's biggest, most neglected health challenge: Non-communicable diseases. Chin J Health Policy.

[6] Guo F, Zhang YH, Wan Q, Zhai TM, Chai BB, Li Y, Wang RR, Huang YX, Chen CM, Li T (2019). Results and analysis of national health accounts in China in 2017. Chin Health Econ.

[7] Zhou ZJ (2016). Supply-side reform: what health care should change. Hosp Manag Forum.

[8] Luo T (2019). How does Britain's general practitioner system play a role in controlling costs. China State Finance.

[9] Schoenmakers B, Buntinx F, Delepeleire J (2009). What is the role of the general practitioner towards the family caregiver of a community-dwelling demented relative?. Scand J Prim Health Care.

[10] Sims J, Kerse NM, Naccarella L, Long H (2008). Health promotion and older people: the role of the general practitioner in Australia in promoting healthy ageing. Aust N Z J Public Health.

[11] Yang H (2019). Declarations from Alma-Ata and Astana: development of general practice is a top priority for achieving universal health coverage. Chin Gen Pract.

[12] National Health Commission of the People's Republic of China. Notice on Issuing Opinions on the Implementation of the Training of Assistant General Practitioners.2016. http://www.nhc.gov.cn/qjjys/s3593/201606/ac7465a778f24fcd9a47f7cec54a3974.shtml. Accessed 16 Jan 2021.

[13] The State Council of the People's Republic of China. Opinions on Deepening the Coordination of Medical Education and Further Promoting the Reform and Development of Medical Education.2017. http://www.gov.cn/zhengce/content/2017-07/11/content_5209661.htm. Accessed 16 Jan 2021.

[14] The State Council of the People's Republic of China. Opinions on Reforming and Improving the Incentive Mechanism for the Training and Use of General Practitioners. 2018.http://www.gov.cn/zhengce/content/2018-01/24/content_5260073.htm. Accessed 16 Jan 2021.

[15] National Health Commission of the People's Republic of China. Notice on the issuance of guidelines for the Establishment of General Medical Disciplines in Standardized Training Bases (General hospitals) for Resident Physicians (trial).2018. http://www.nhc.gov.cn/qjjys/s3593/201809/951a65647c41459b858ccf1c26fc1acb.shtml. Accessed 16 Jan 2021.

[16] Theodorakis PN, Mantzavinis GD, Rrumbullaku L, Lionis C, Trell E. Measuring health inequalities in Albania: a focus on the distribution of general practitioners. Hum Resour Health. 2006; 4(1). 10.1186/1478-4491-4-5 PMC1395320 16504028

[17] Shannon GW, Cutchin MP (1994). General practitioner distribution and population dynamics: Munich, 1950–1990. Soc Sci Med.

[18] Wilkinson D, Symon B (2010). Inequitable distribution of general practitioners in Australia: estimating need through the Robin Hood Index. Aust N Z J Public Health.

[19] Johnston G, Wilkinson D (2001). Increasingly inequitable distribution of general practitioners in Australia, 1986–96. Aust N Z J Public Health.

[20] Hu WP, Yang J, Xu RL, Ni R, Meng XL, Yu EY (2015). Equity analysis of the allocation of general practitioners in Mainland Ghina. Chin Gen Pract.

[21] Huang Fu HH, Li HY (2018). Research on equity of resource allocation of general practitioners in China based on the Theil index and grey prediction method. Health Econ Res.

[22] Zhou LL, Wang HP, Xie L, Ding H (2017). Current situation and equity analysis of general practitioners' resource allocation in China. Chin Gen Pract.

[23] Zhou HL, Lan XJ, Si MS, Li ZF, Li SX (2018). Analysis on equity of general practitioners allocation in guangxi based on concentration index and Gini Coefficient. Chin Health Econ.

[24] Cheng YM, Xu YF, Wen N, Ma X, Wang F (2020). Distribution equity and development trend of general practitioners in Shandong Province during 2013–2016. Chin Gen Pract.

[25] Lin CM, Zhang X, Yang XQ, Qin JM (2016). Allocation differences and fairness of community general practitioners in districts and counties of Beijing from 2011 to 2014. Chin Gen Pract.

[26] Liang LN, Xie Y (2011). Gini coefficient and its application expansion. J Qiqihar Univ (Philosophy & Social Science Edition).

[27] Li ZY, Kong XJ, Ren R (2013). Research on the development trend of equity in health resource allocation in Liaoning province: based on Gini coefficient and Theil index. Chin Health Econ.

[28] Yuan WW, Wei FQ, Liu WW, Zhang Z, Ma J (2015). A methodological study on the evaluation of equity in health resource allocation by agglomeration analysis. Chin Hosp Manag.

[29] National Bureau of Statistics of China. China Statistical Yearbook 2013. http://www.stats.gov.cn/tjsj/ndsj/2013/indexeh.htm. Accessed 16 Jan 2021.

[30] National Bureau of Statistics of China. China Statistical Yearbook 2014. http://www.stats.gov.cn/tjsj/ndsj/2014/indexeh.htm. Accessed 16 Jan 2021.

[31] National Bureau of Statistics of China. China Statistical Yearbook 2015. http://www.stats.gov.cn/tjsj/ndsj/2015/indexeh.htm. Accessed 16 Jan 2021.

[32] National Bureau of Statistics of China. China Statistical Yearbook 2016. http://www.stats.gov.cn/tjsj/ndsj/2016/indexeh.htm. Accessed 16 Jan 2021.

[33] National Bureau of Statistics of China. China Statistical Yearbook 2017. http://www.stats.gov.cn/tjsj/ndsj/2017/indexeh.htm. Accessed 16 Jan 2021.

[34] National Bureau of Statistics of China. China Statistical Yearbook 2018. http://www.stats.gov.cn/tjsj/ndsj/2018/indexeh.htm. Accessed 16 Jan 2021.

[35] China National Health and Family Planning Commission (2013). China health statistical yearbook 2013.

[36] China National Health and Family Planning Commission (2014). China health statistical yearbook 2014.

[37] China National Health and Family Planning Commission (2015). China health statistical yearbook 2015.

[38] China National Health and Family Planning Commission (2016). China health statistical yearbook 2016.

[39] China National Health and Family Planning Commission (2017). China health statistical yearbook 2017.

[40] China National Health and Family Planning Commission (2018). China health statistical yearbook 2018.

[41] Mao Y, Liu JL, Yang J, Xu F, Zhang MJ (2013). Analysis on fairness of China's human resource allocation for health in 2011. Chin Health Econ.

[42] Organization PAH (2001). Measuring health inequalities: Gini coefficient and concentration index. Epidemiol Bull.

[43] Yuan SW, Wei FQ, Liu WW, Zhang Z, Ma J (2015). Methodology discussion of health resource allocation equity evaluation based on agglomeration degree. Chin Hosp Manag.

[44] Xie HK, Wang ZF, Li NY (2017). Research on the correlation between occupational stress coping style and physical and mental health of general practitioners. Occup Health.

[45] Li SJ, Zhang HR, Zhu LN, Qin Y, Ma WY, Ma L (2015). Study of general practitioners' work pressure and work satisfaction status and its influencing factors. Chin Gen Med.

[46] Zheng YL, Yu F, Chen YL, Yu MY, Liu L, Gan Y, Li LQ, Yang YD, Lu ZX (2019). Occupational burnout of general practitioners in China and its influencing factors. Chin Gen Pract.

[47] Meng XM, Pan XY, Dong Q (2013). The comparative study on general practitioner training the at home and abroad. Hebei Med J.

[48] Yao DP. Research on Urban Family Doctor Service Capacity Building under the Background of the Internet [D]. University of Science and Technology of China, 2017.

[49] Hu MD (2016). Exploration and practice of internet + community health. Shanghai Pharm.

[50] Hao Y, Wang L, Liu XY, Yu HY, Jia HY, Guo XL (2018). Research on IFOC model-based community home-based care service model in Fangzhuang Community Health Service Center. Chin Gen Pract.

[51] Wang X, Yu CZ, Wang XW, Wang JJ (2014). Analysis on the status and equity of human resource allocation in community health services in Guangdong Province. Chin Gen Pract.

[52] Zhao B, Yang LW, Zheng N (2014). The equity analysis of urban community health resource allocation based on Gini Coefficient and Theil Index. Chin Gen Pract.

[53] Liu Y, Jiang GP, Ren JJ (2019). Status quo and development strategy of general practitioners training in China. China Eng Sci.

[54] Zeng YX (2018). Talent is the first resource. Health Vision.

